# Neighborhood socioeconomic deprivation and physical activity associate with extracellular vesicle size and cargo in a community-based cohort of women

**DOI:** 10.1038/s41598-026-49679-6

**Published:** 2026-04-28

**Authors:** Yvonne Baumer, Elizabeth M. Aquino Peterson, Matthew R. Bavuso, Noel Miller, Cristhian A. Gutierrez-Huerta, Nithya P. Vijayakumar, Sam J. Neally, Kaveri Curlin, Valerie M. Mitchell, Billy S. Collins, Neelam R. Redekar, Subrata Paul, Anca D. Dobrian, Tiffany M. Powell-Wiley

**Affiliations:** 1 Social Determinants of Obesity and Cardiovascular Risk Laboratory, National Heart Lung and Blood Institute, National Institutes of Health, Bethesda, MD USA; 2 Department of Biomedical and Translational Sciences, Eastern Virginia Medical School at Old Dominion University, Norfolk, VA USA; 3 Integrative Data Sciences Section, National Institute of Allergy and Infectious Diseases, National Institutes of Health, Bethesda, MD USA; 4 Intramural Research Program, National Institute on Minority Health and Health Disparities, National Institutes of Health, Bethesda, MD USA

**Keywords:** Social Determinants of Health, Cardiovascular disease, Extracellular vesicles, miRNA, Endothelium, Physical activity, Cardiovascular biology, Socioeconomic scenarios

## Abstract

African American (AA) women face disproportionate cardiovascular disease (CVD) risk due to adverse social determinants of health (SDoH), including lower socioeconomic status (SES). Physical activity (PA) and plasma extracellular vesicles (EVs) modulate CVD risk, but their relationships with SDoH remain unclear. This study examines associations between SDoH, PA, and plasma EVs in a Washington, DC-based cohort of at-risk AA women. Participants (*N* = 24, Age: 57 ± 12, BMI: 35 ± 6, ASCVD: 9 ± 5) joined the Step It Up Community-Engaged, Digital Health Physical Activity Intervention pilot study. EVs were isolated from fasting plasma samples. Multivariable regression, adjusted for BMI and ASCVD 10-year risk, showed that neighborhood socioeconomic deprivation (NSD) was associated with decreased EV size (β=-0.49, *p* = 0.01), while higher daily step count was associated with increased EV size (β = 0.48, *p* = 0.02). EV miRNA cargo, including miR-1246, miR-28-5p, and miR-765, showed distinct expression patterns with EV size, NSD, and PA. In vitro, endothelial cell (EC) barrier integrity directly correlated with miR-28-5p (*r* = 0.73, *p* = 0.007) and miR-765 (*r*=-0.58, *p* = 0.049). EC migration correlated negatively with miR-28-5p (*r*=-0.67, *p* = 0.02). Findings highlight smaller EV size associated with high NSD and low PA, suggesting miR-28-5p may be a biomarker or mediator for CVD pathogenesis. PA interventions may mitigate adverse SDoH effects and establish EV miRNA cargo as biomarkers and/or intervention mediators.

**Supplementary Information:**

The online version contains supplementary material available at 10.1038/s41598-026-49679-6.

## Introduction

Social determinants of health (SDoH) comprise socioeconomic, environmental, and psychosocial factors impacting an individual’s daily life. In a recent review, we published an updated framework summarizing various SDoH domains ranging from structural inequalities (e.g., socioeconomic status [SES], neighborhood-level deprivation due to redlining and discriminatory policies) to individual-level stressors (e.g., perceived experiences of racism or discrimination, chronic stress, and violence).^1^ The life-long experience of adverse SDoH has been linked to obesity, diabetes, and subsequent cardiovascular disease (CVD).^2,3^ This excess risk for cardiovascular (CV) risk factors and CVD itself disproportionally affects minoritized populations and socioeconomically under-resourced communities.^3,4^.

African Americans (AA) living in lower-resourced neighborhoods have increased CVD mortality risk^5^, emphasizing the relationship between the neighborhood social environment and CVD outcomes.^6^ From 2015 to 2018, the rate of CVD was most prevalent in the non-Hispanic Black population in the United States, with 58.8% of non-Hispanic Black women affected.^7^ Recent studies have demonstrated that neighborhood socioeconomic deprivation (NSD) as a marker of neighborhood social environment is an independent predictor of dyslipidemia, hospitalized heart failure risk, and diabetes risk.^8–10^ Additionally, non-Hispanic Black women living in socially vulnerable neighborhoods have a greater likelihood of CVD-related risk factors, including hypertension, diabetes, and obesity, compared to those living in less vulnerable neighborhoods.^11,12^.

The effects of chronic stress, especially related to the neighborhood level, on cellular signaling pathways and overall health are not yet fully understood and require further research.^13^ One way to examine the biological effects of SDoH is via extracellular vesicles (EVs), vessels for the body to relay information between the brain and the body’s periphery.^14^ EVs are lipid bilayer nanoparticles in the circulatory system that deliver biological cargo from one cell to another.^15,16^ EVs can be released from and taken up by various cells within the body and are involved in intercellular communication.^17^ EVs are highly heterogeneous in size and their molecular cargo (e.g., protein, lipids, or microRNA [miRNA]). EV’s diversity of cargo enables a wide range of biological effects to be delivered to recipient cells. Prior studies suggest EVs serve as disease biomarkers, potentially allowing better prediction and diagnosis of hypertension, diabetes, obesity, and CVD.^17–21^ Studies demonstrate a connection between chronic stress and EVs in animals.^22,23^ However, fewer studies investigate this connection in humans.^24^ Data from the Healthy Aging in Neighborhoods of Diversity Across the Life Span (HANDLS) study showed that the association between EV inflammatory proteins and mortality was significantly higher in individuals living below the poverty line^25^, and EV concentration, size, and cargo differ across age, sex, and gender groups.^26^ Racial differences have been observed in EV cargo among hypertensive AA and Caucasian women.^27^.

Physical activity (PA) has an established role in improving mental and physical well-being. Promoting PA, independent of weight loss, has been found to benefit individuals and lower CVD risk.^28^ However, little research examines PA as an intervention tailored for under-resourced populations. Likewise, AA women face intrapersonal barriers to PA, such as lack of time, physical appearance concerns, financial barriers, and environmental barriers, such as lack of sidewalks and safety.^29^ Evidence indicates an essential impact of PA on EVs. EVs are known to play a role in skeletal muscle adaption to exercise and are induced by PA.^30,31^ Endurance exercise in males was associated with differences in protein cargo.^32^ In adolescent study participants with obesity, PA changes were associated with differences in EV concentration, size, and protein levels.^33^ EV-associated miRNAs have been linked with different types and intensities of exercise.^34^ However, more research is needed to fully understand the mechanisms and potential therapeutic applications of exercise-induced EVs.

Currently, the research linking EVs to SDoH, especially NSD, and its relationship with EV characteristics in populations at the highest risk for exposure to adverse SDoH and subsequent CV risk is still in its early stages. Emerging evidence suggests that EVs may play a significant role in mediating the effects of social determinants on health outcomes. Therefore, this study addresses a critical gap in the understanding of CVD risk factors among AA women from under-resourced communities. By examining the relationships between NSD, PA, and EV characteristics in this high-risk population, we aimed to uncover novel biological mechanisms underlying health disparities. Our comprehensive approach, combining community-based sampling with advanced EV and miRNA profiling, provides unique insights into how SDoH may influence CV risk at the molecular level. Furthermore, through in vitro experiments, we determined the impact of the isolated EVs and their cargo on endothelial function, a hallmark of CVD development and progression.^35^.

## Results

The study cohort was a convenience sample of AA women with CVD risk (*N* = 24, Table 1) with a mean age of 57 ± 12 years and class I obesity. Participants were considered at intermediate risk for ASCVD within the next ten years at the time of data collection (mean ASCVD 10-year risk score: 8.65 ± 5.58).

**Table 1:** Baseline characteristics of Pilot Study for the Step It Up Physical Activity Mobile App tailored to Neighborhood Intervention Study, 2018/2019.

	Pilot study (*n* = 24)^a^
Demographics and medical history	
Age, years	56.96 ± 12.38
Female, N (%)	24 (100)
Race, African American, N (%)	24 (100)
Body mass index, kg/m2	34.77 ± 6.25
Hypertension Prevalence, N (%)	11 (45.83)
Hyperlipidemia Prevalence, N (%)	6 (25)
Diabetes Prevalence, N (%)	2 (8.33)
ASCVD 10-year Risk Score ^b^	8.65 ± 5.58
Social Determinants of Health (SDoH) measures	
Neighborhood Socioeconomic Deprivation (NSD)^^^	− 0.48 ± 3.92
Individual-Level Socioeconomic Status (SES)^‡^	73.64 ± 23.00
Physical Activity Measures (Objective and Self-Reported)	
Daily Step Count	8905.69 ± 3766.42
Self-reported Leisure Activity, min/week	365.00 ± 355.84
Self-reported Moderate Activity, min/week	293.75 ± 362.41
Self-reported Vigorous Activity, min/week	163.75 ± 187.19
Endothelial Extracellular Vesicles Parameters and Function	
EV concentration (EV-c), number/ml	1.81 × 10^+ 11^ ± 9.50 × 10^+ 10^
EV size (EV-z), nm	82.48 ± 14.05
EV-mediated EC barrier integrity, “Delta” TEER AUC	24.33 ± 1.53
EV-mediated Wound Healing, “Delta” TEER AUC	43.78 ± 1.82

^a^Values reported in the table as Mean ± Standard Deviation for parametric continuous variables, Median (Interquartile Range) for nonparametric continuous variables, and N (%) for categorical variables.

^b^Atherosclerotic Cardiovascular Disease (ASCVD) 10-year Risk Score.

^^^NDI - neighborhood deprivation index derived from U.S. Census data (higher score=more socioeconomically deprived neighborhood).

^‡^SES – individual-level socioeconomic status (higher value=higher self-reported household income).

### EV size associates with neighborhood socioeconomic deprivation as well as daily step count

**Fig. 1:**
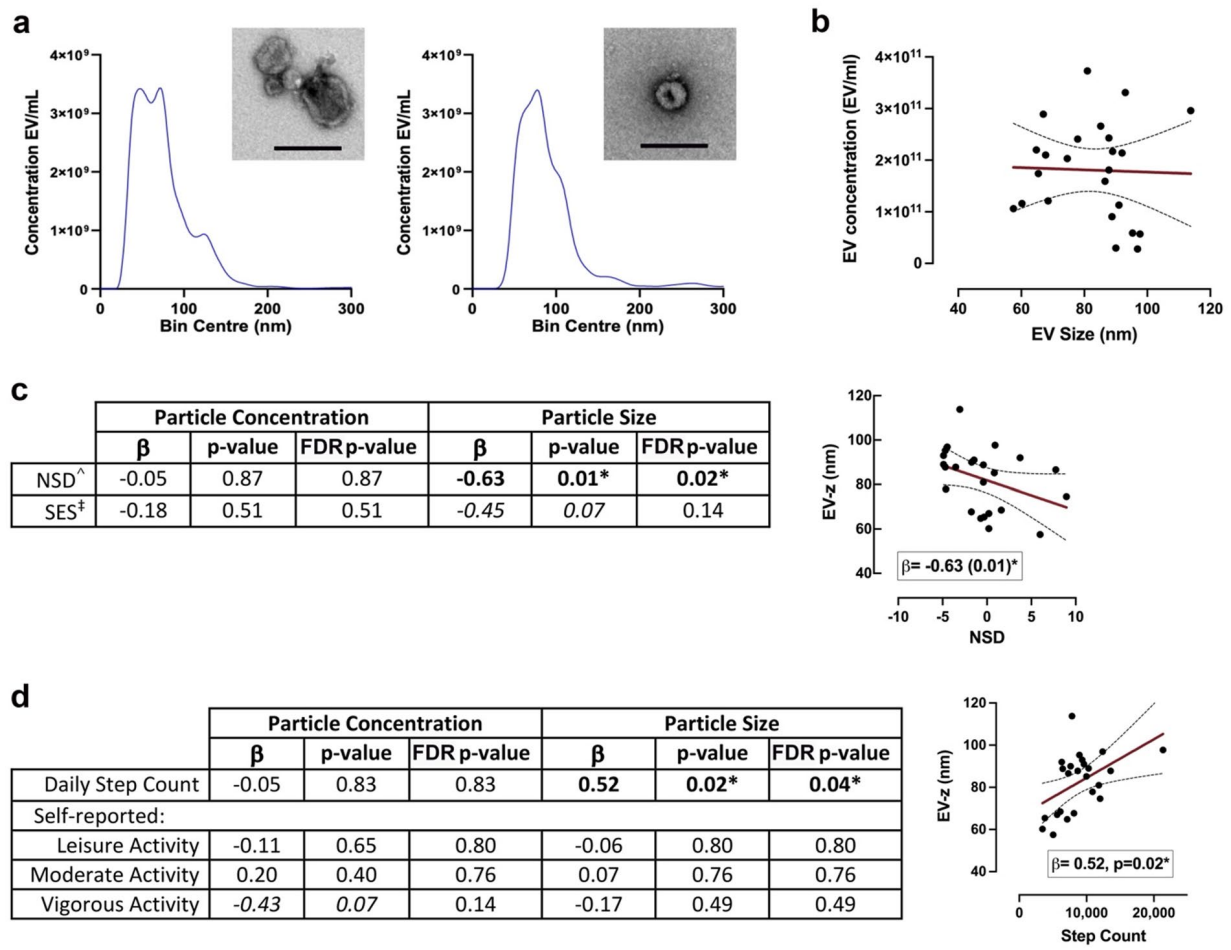
EV size associates with Neighborhood Socioeconomic Deprivation (NSD) and Daily Step Count. (**a**) Representative EV size distribution profile and respective EV morphology using TEM negative staining TEM images were taken at 30,000x magnification (scale bar: 100 nm). (**b**) No association was found between size and concentration of plasma EVs isolated from 24 study participants. (**c**) Multivariable linear regression modeling of associations between measures of SDoH and EV concentration or size. Models are adjusted for ASCVD 10-yr risk score and BMI. Findings are displayed as standardized beta coefficients followed by the unadjusted p-value and the FDR adjusted p-value. Visual representation of the association between NSD and EV size (EV-z). (**d**) Multivariable linear regression modeling of associations between objective as well as self-reported measures of physical activity and EV concentration or size. Models are adjusted for ASCVD 10-yr risk score and BMI. Findings are displayed as standardized beta coefficient followed by the unadjusted p-value and the FDR adjusted p-value. Visual representation of the association between NSD and EV size (EV-z). (Significance is indicated by * and bold font with a p-value < 0.05; EV-c – Extracellular vesicle concentration, EV-z – extracellular vesicle size, ^NSD-neighborhood socioeconomic deprivation, SES-individual level socioeconomic status)

EVs were isolated from study participants’ fasting heparin plasma samples and characterized for their size distribution and concentration. Figure 1a shows two examples of EV profile and their respective negative staining electron microscopy representative micrographs. The profiles of all 24 study participants are given in Supplementary Fig. 1. The average EV concentration for the study participants was 1.81 × 10^+ 11^ EVs/mL, and their average size was 82.5 nm (Table 1). EV size and EV concentration were not associated with one another (Fig. 1b).

Given the scarce data regarding the relationships between SDoH and circulating plasma EVs, our initial investigation aimed to assess how neighborhood socioeconomic deprivation (NSD) and individual-level SES may associate with EV concentration and size (Fig. 1c). NSD inversely associated with EV particle size after adjustment for BMI and ASCVD risk score (β=-0.63, *p* = 0.01, Fig. 1c). This association remained significant even after adjustment for BMI and ASCVD risk score (padj = 0.02). In contrast, individual-level SES was not associated with EV particle size. No significant associations of NSD or SES with EV concentration were found (Fig. 1c).

In the second step, we aimed to determine if physical activity (PA) was associated with EV characteristics (Fig. 1d). Baseline daily step count as measured by Fitbit, an objective measure of PA, positively associated with EV size (β = 0.52, *p* = 0.02, Fig. 1d), which remained significant after FDR adjustment (padj = 0.04). No significant association was found with EV concentration. Self-reported measures of PA were not associated with either EV concentration or size.

### miRNA content of EVs varies by EV size, NSD, and daily step count

**Fig. 2:**
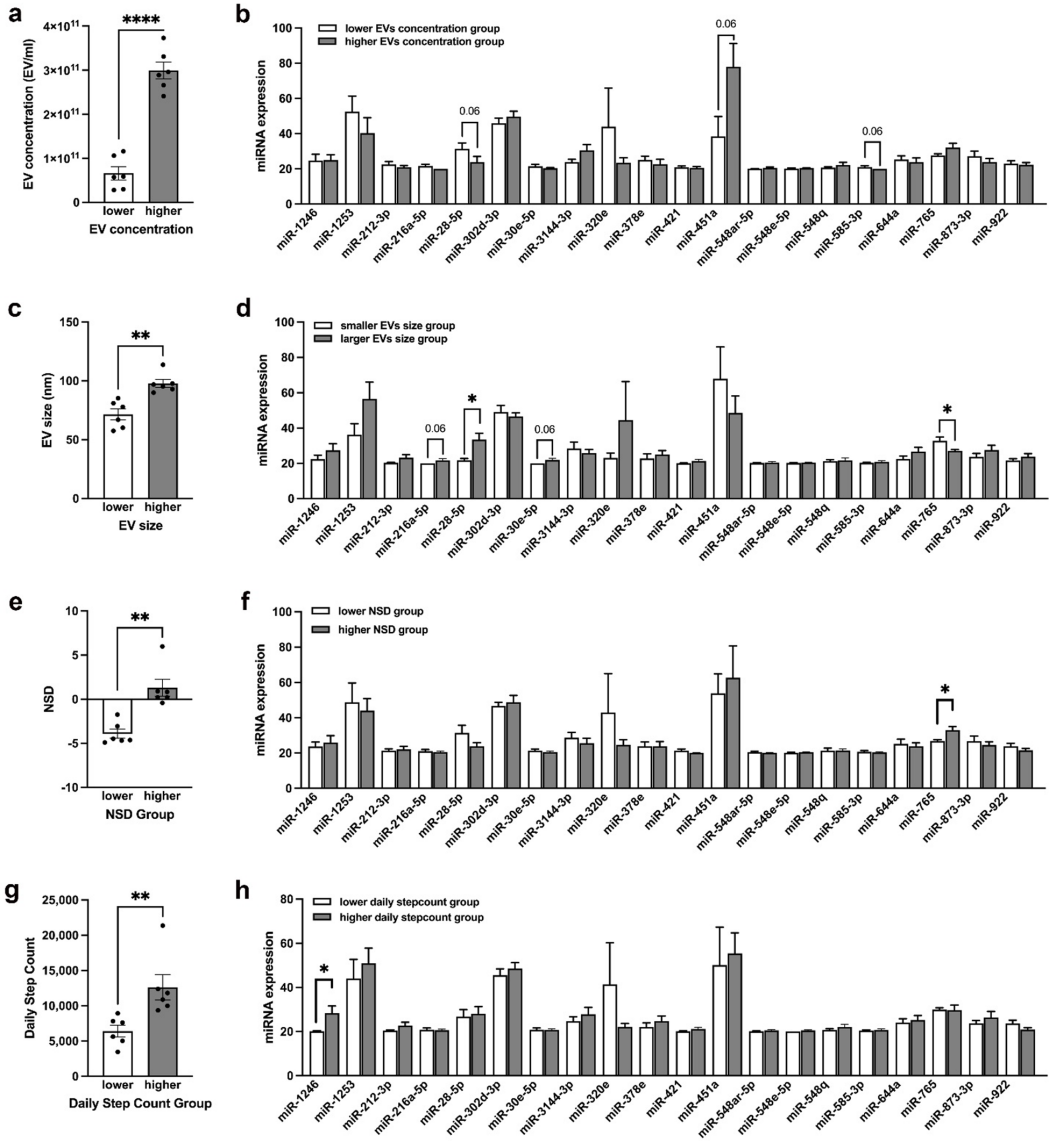
miRNA content in EVs varies by EV size, NSD, and Daily Step count. From the initial cohort of 24 donors, we selected 12 donors who exhibited diverse profiles in terms of EV concentration, EV size, NSD, and daily step count. Donor selection was based on upper and lower quantiles (*n* = 6) of EV size ranking. We then analyzed the miRNA content within the EVs from these selected donors. Bivariate comparisons (Mann-Whitney test) of detected miRNAs by EV concentration (**a**/**b**), EV size (**c**/**d**), NSD (**e**/**f**), and Daily Step Count (**g**/**h**) are displayed. (Significance is indicated by * and with a p-value < 0.05, while ** indicates a p-value < 0.001; p-values indicate raw unadjusted p-values; error bar indicates standard error of mean. NSD=neighborhood socioeconomic deprivation)

We analyzed the miRNA cargo of EVs from 12 study participants who exhibited significant differences in EV concentration and size, NSD, and daily step count (Fig. 2a, c, e, g). Overall, 20 miRNAs were detectable within the EVs. For each miRNA, bivariate comparisons by EV concentrations, EV size, NSD, and daily step count were performed (Fig. 2b, d, f, h).

A comparison of EV miRNA content by EV concentration (Fig. 2a/b) did not reveal significant differences in miRNA cargo. However, miR-28-5p, miR-451a, and miR-585-3p trended to be differentially expressed.

Secondly, comparing miRNA content in EVs by EV size (Fig. 2c/d) revealed that miR-28-5p was expressed at higher levels in the larger EV size group, while miR-765 expression was lower in the larger EV size group. Two additional miRNAs displayed trends to differential expression by EV size: miR-216a-5p and miR-30e-5p.

When comparing miRNA content by NSD groups, miR-765 displayed increased expression in EVs of study participants residing in more highly deprived neighborhoods (Fig. 2e/f). In a comparison by daily step count (Fig. 2g/h), miR-1246 shows increased expression in study participants with higher daily step counts.

### EV miR-28-5p and miR-765 content correlates with EV-induced changes in endothelial function

**Fig. 3:**
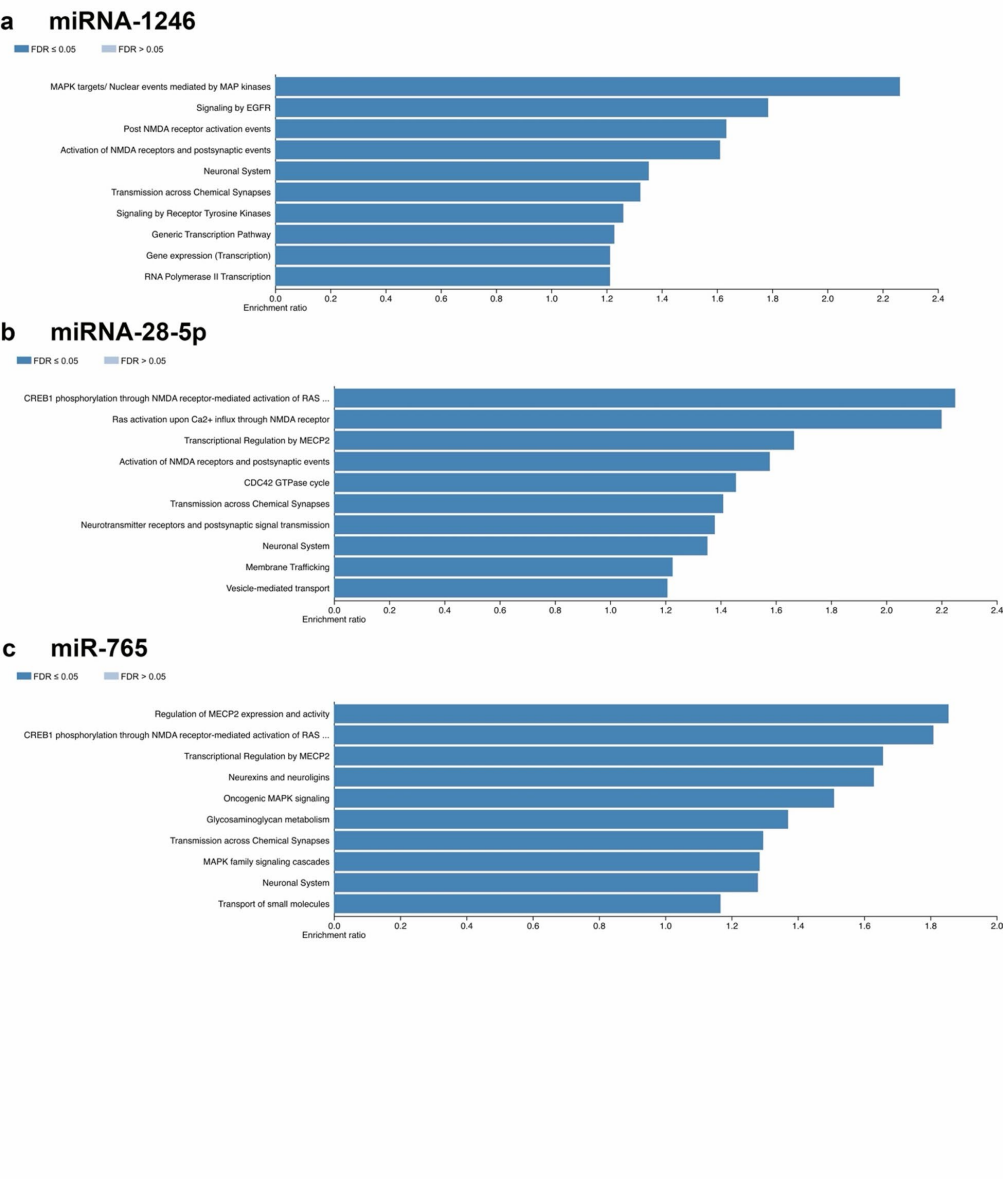
Bar graph of enriched pathways shown to be significantly impacted by the indicated miRNAs in an unbiased biostatistical analysis approach. The three miRNAs shown to be significantly different in EVs by either EV size, NSD, daily step count, or a combination of these factors are miR-1246 (**a**), miR-28-5p (**b**), and miR-765 (**c**). The x-axis indicates the enrichment ratio, while the y-axis indicates the signaling pathway identified.

Bivariate comparisons indicated significant differences by PA, NSD, or EV size for miR-1246, miR-28-5p, and miR-765. Therefore, we employed an overrepresentation analysis of miRNA gene targets of these three miRNAs. Reactome pathways identified to be significantly affected by the three miRNAs with an FDR *p* ≤ 0.05 are summarized in Fig. 3 and Supplementary Table 1. These pathways highlight signaling processes that show relevant pathogenic effects in various pathologies, including CVD. In particular, pathways related to MAP kinase-related signaling, EGFR signaling, CDC42 GTPase cycle, MECP2 regulation, as well as CREB1 phosphorylation have been implicated in CVD pathologies.^36–40^ We then focused on the potential impact of EVs on the endothelium, as it is known that plasma circulating EVs first encounter and are taken up by ECs.^41^.

**Fig. 4:**
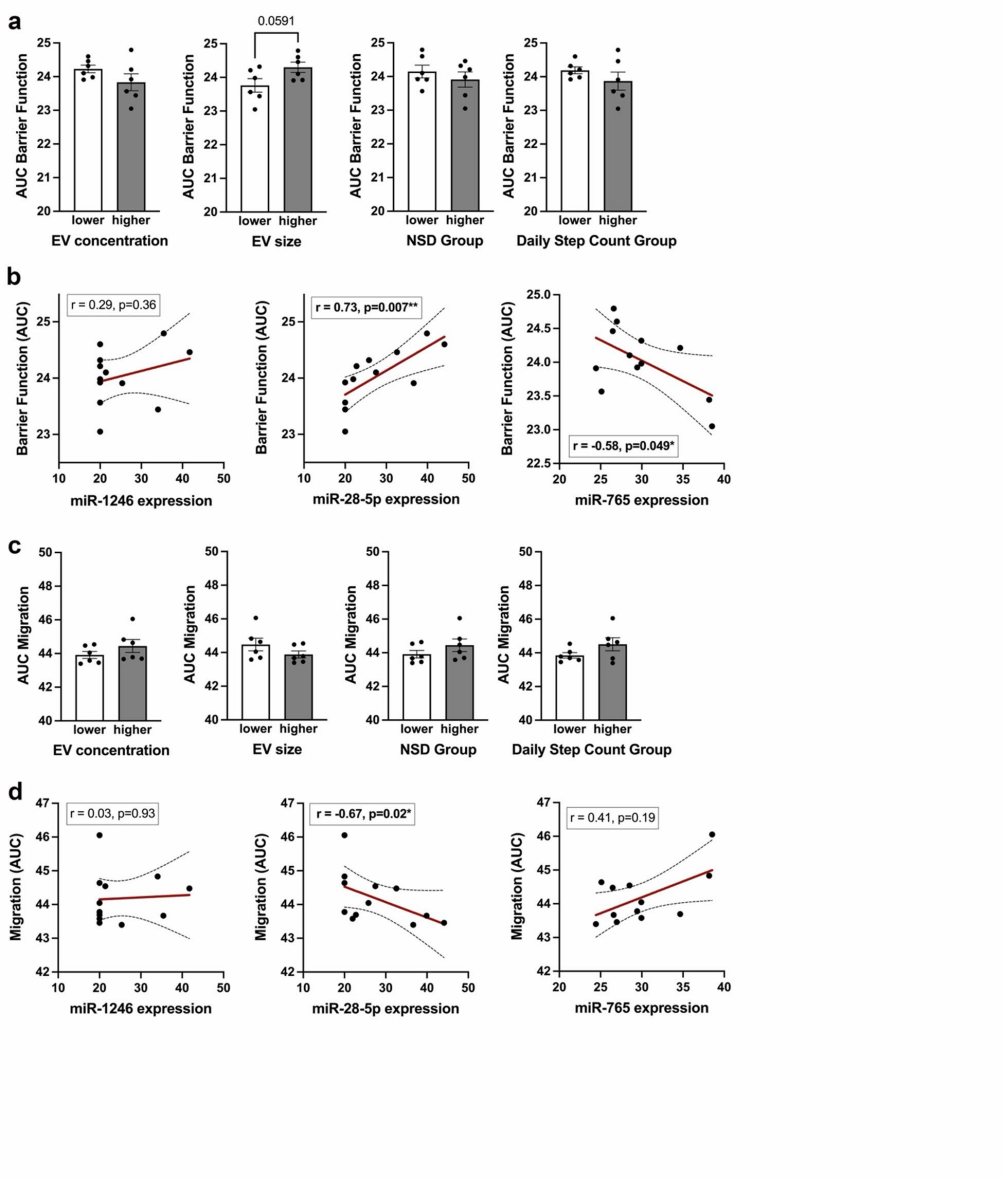
EV miR-28-5p expression is associated with increasing endothelial barrier function and decreasing EC migration. Human coronary artery endothelial cells (HCAECs) cultured on ECIS electrodes were treated with EVs isolated from human plasma. For the first 24 h of treatment, endothelial barrier function was measured. After 24 h of treatment, the monolayer was wounded utilizing the ECIS wounding module. Subsequently, the migration of HCAECs into the wounded area was measured. (**a**) Bivariate comparison of barrier function by EV concentration and size, NSD, and daily step count. (Mann Whitney test, *n* = 6 each group) (**b**) Correlation analysis of EV miRNA content to EC barrier function is plotted with the correlation coefficient and the p-value indicated. (*n* = 12, bold font indicates significance with a p-value < 0.05, significant p-values are further indicated by the *) (**c**) Bivariate comparison of migration by EV concentration and size as well as NSD and daily step count. (Mann Whitney test, *n* = 6 each group) (**d**) Correlation analysis of EV miRNA content to EC barrier function is plotted with the correlation coefficient and the p-value indicated. (*n* = 12, bold font indicates significance with a p-value < 0.05, significant p-values are further indicated by the *, while ** indicates a p-values < 0.001; p-values indicate raw unadjusted p-values) D’Agostino & Pearson test was performed for all datasets. Normally distributed data were subjected to Pearson correlation, while Spearman correlation was utilized if the data did not follow a normal distribution.

Endothelial barrier function was recorded for 24 h following incubation with individual study participants’ EVs and shown as AUC (Supplementary Fig. 2a). No significant differences were found when comparing barrier function by particle concentration, NSD, or daily step count; comparison by EV particle size trended to significance with tighter barrier function in the larger EV group (Fig. 4a). We found a positive correlation between the presence of miR-28-5p in EVs and increased endothelial barrier function (*r* = 0.73, *p* = 0.007, FDR *p* = 0.021), while EV content of miR-765 was negatively correlated with endothelial barrier function (*r*=-0.58, *p* = 0.049, FDR *p* = 0.074) (Fig. 4b), which was attenuated after adjusting for multiple comparison. miR-1246 content did not correlate significantly with EC barrier function (Fig. 4b).

The impact of EVs on EC migration was recorded for an additional 24 h (Supplementary Fig. 2c). No significant differences by group could be determined when comparing EC migration after EV treatment by either EV concentration or size and NSD or daily step count (Fig. 4c). However, when performing correlation of EV miRNA content, miR-28-5p levels correlated negatively with EC migration (*r*=-0.67, *p* = 0.02, FDR *p* = 0.06), while miR-1246 and miR-765 did not display any correlations (Fig. 4d). Taken together, our data suggest that EV miRNA content may alter EC function.

### miR-1253, miR-28-5p, and miR-922 are differentially expressed in EVs, impairing either EC integrity or migration

Lastly, we determined EV cargo differences by their impact on EC barrier function or endothelial migration (Supplementary Fig. 2). For the initial 24 h, EC barrier function was recorded. After 24 h, the endothelial layer was injured, and subsequent migration of EV-treated HCAECs was recorded for an additional 24 h.

We analyzed the AUC for endothelial barrier function from each donor’s EV treatment (Supplementary Fig. 2). This analysis involved comparing the miRNA contents in EVs in a bivariate manner. Our findings indicated that miR-1253 and miR-28-5p were more highly expressed in EVs, which correlated with enhanced barrier integrity. Similarly, miR-644a showed a tendency toward higher expression in EVs linked with stronger barrier function, whereas miR-3144-3p tended to be less expressed in this group. Additionally, when we categorized the results of EC migration into two groups (Supplementary Fig. 2) and compared their cargo in a bivariate analysis (Supplementary Fig. 2), miR-922 varied significantly between the groups. The Reactome pathways potentially regulated by these miRNAs are summarized in Supplementary Fig. 3 and Supplementary Table 2. Pathway enrichment analysis of the target genes impacted by the five miRNAs identified in our studies highlights ten significantly affected Reactome pathways (FDR *p* ≤ 0.05), known to be associated with processes or diseases related to EC dysfunction (Supplementary Fig. 3 and Supplementary Table 3).

## Discussion

In the pilot study of the Step It Up Community-Engaged, Digital Health Physical Activity intervention, we evaluated the relationships between NSD and PA with EV concentration, size, and miRNA cargo in AA women living in under-resourced Washington, DC area neighborhoods. To our knowledge, our study is a first of its kind to examine associations between adverse SDoH, PA levels, and EV characteristics and cargo. Furthermore, our study focuses on AA women, a group at the highest risk for obesity and CVD and traditionally under-represented in research and clinical trials.^42,43^.

Our findings reveal statistically significant associations between research was supported [in partNSD, PA, and EV characteristics. Residents of more socioeconomically deprived areas exhibited smaller EVs with higher miR-765 expression. Conversely, higher daily step counts are associated with larger EVs and elevated miR-1246 expression. We identified two miRNAs differentially expressed based on EV size: miR-28-5p showed higher expression in larger EVs, while miR-765 was more prevalent in smaller EVs. Both miR-28-5p and miR-765 EV contents were associated with changes in endothelial barrier integrity when endothelial monolayers were exposed to participants’ EVs. Further analysis of endothelial barrier integrity and migration revealed differential expression of miR-1253, miR-922, and miR-28-5p, highlighting their potential role in endothelial barrier integrity and migration. Interestingly, miR-28-5p expression was implicated in several of our findings and could serve as a possible target of interest for future studies of EV-induced changes in endothelial barrier function.

We propose that residents of socioeconomically disadvantaged areas produce smaller EVs containing specific miRNAs: miR-28-5p and miR-765. These EVs, when taken up by endothelial cells, appear to positively alter endothelial barrier function, a critical factor in vascular health.^44^ This EV-mediated pathway may represent a key molecular link between SDoH and cardiovascular outcomes. Additionally, our data suggest that PA could mitigate the impact of NSD on EV size and content and potentially EV-mediated endothelial dysfunction.

Prior studies have examined the relationships between socioeconomic factors and EV markers. Data collected from the HANDLS study did not reveal any significant associations between EV concentration and poverty status or ethnicity.^25^ However, living below the poverty line was associated with significantly higher expression of EV inflammatory proteins (e.g., CST5, CXCL10, VEGF-A). A review article summarizing the literature on race and ethnicity in relation to EVs reported no differences in EV concentration or size when comparing AA to Caucasian study participants but differences in cargo.^26^ However, differences in EV cargo were reported by ethnicity or race in patients with cancer.^37^ None of these studies accounted for SDoH and their potential impact on EV markers.

While direct evidence linking NSD to EVs is limited, there is a growing body of research that suggests potential connections through various biological pathways. For example, sleep deprivation occurs with higher frequency in socioeconomically deprived neighborhoods and has undoubtedly been linked to an increased risk of CVD.^45^ One night of sleep deprivation increased the release of small EVs in healthy individuals^46^, potentially providing a pathway for how neighborhood deprivation could impact EVs.

Only a few studies explored the impact of exercise and PA on EV size and/or concentration in humans with limited consensus. Previous work in humans shows that the benefits of PA may be driven, in part, by EVs released into circulation during exercise.^47^ In pregnant patients^48^, the concentration of small EVs increased significantly, with no difference in mean vesicle size post-exercise.^49^ It has been reported that exercise training did not alter endothelial EV concentration in post-menopausal women.^50^ Acute endurance exercise increased the concentration of small EVs (30–150 nm) in plasma, with a shift towards smaller EVs post-exercise.^34^ Heavy resistance exercise reduced EV size in men but not women.^51^ In young individuals living with obesity, 6 weeks of resistance training was associated with increased EV concentration independent of changes in insulin sensitivity.^33^ However, in individuals displaying improvement of insulin sensitivity (responders), an increase of EV size was observed when compared to the non-responder group.^33^ However, other studies did not detect significant changes in EV size (reviewed in, for example, after two consecutive bouts of muscle-damaging exercise).^52^ These studies suggest that PA can indeed influence EV size, with a general trend towards increased release of smaller EVs in response to exercise. However, the specific effects appear to depend on factors such as exercise intensity, duration, and modality. Our study, however, shows that baseline PA was associated with larger EV size. The potential difference in our data could lie in the intensity of PA investigated. PA interventions have focused on high-intensity interventions with blood draws and immediate post-exercise EV characterization, which likely differs from the effects of long-term, lower-intensity PA at baseline (i.e., walking) on the human body. In general, there is little mechanistic explanation for small EV pathogenicity. Potential mechanisms include more efficient transfer of bioactive molecules by smaller EVs due to their higher surface-to-volume ratio and the curvature of smaller EVs, which can affect the presentation of surface proteins, potentially altering their interactions with target cells.^53,54^ Our findings did not show any associations between baseline daily step counts and plasma EV concentration. Several factors may account for these divergent findings, including daily step counts as a single variable compared to more structured exercise interventions from other studies. Other factors may include variations in EV isolation techniques, our focus on total plasma EV counts rather than cell-specific subpopulations, and our participant demographics as well as our relatively small sample size.

The impact of PA on EV concentration and size remains inconsistent across studies, largely due to variations in key research parameters.^55^ Regardless of methodological differences, studies consistently demonstrate that PA modifies EV cargo, promoting anti-inflammatory^56^, anti-oxidative^57^, and cardioprotective properties.^32^ Despite variability in physical EV characteristics, this convergence in functional outcomes underscores the importance of considering multiple aspects of EV biology when assessing the impact of PA. It also suggests the beneficial effects of exercise may be mediated more through alterations in EV cargo rather than changes in EV size or concentration alone.

In our study, we investigated miRNA cargo and found five miRNAs of potential importance: miR-765, miR-1246, miR-1253, miR-922, and miR-28-5p. Each of these miRNAs have been connected to CVD risk. For example, circulating levels of miR-765 were increased in patients with coronary artery disease^58^ and is increased in women with arterial stiffness.^59^ Serum and plasma miR-1246 may be potential biomarkers to diagnose hypertension^60^ and stroke^61^ given they were highly expressed in these disease states. Overexpression of miR-1253 promoted apoptosis of vascular smooth muscle cells, contributing to atherosclerosis^62^ and may contribute to racial differences in hypertension between AA and Caucasian women.^63^ Dysregulation of miR-922 has been associated with the pathogenesis of acute myocardial infarction.^64^ Interestingly, pathway analysis of the overlapping target genes of these five miRNAs highlights MAPK signaling and regulation of MECP2, an important regulator of epigenetic gene expression. Both of these important processes have, in the past been linked to one another^65^, to endothelial dysfunction, atherogenesis^66^, and chronic stress.^67^.

Our analysis revealed EV-associated miR-28-5p as a potential player in the complex interplay between EV characteristics and endothelial function. This miRNA emerged as a standout candidate due to its consistent differential expression across two critical parameters: EV size and endothelial barrier integrity. In our dataset, elevated EV miR-28-5p expression is correlated with endothelial barrier integrity. It appears best characterized by its importance in reverse cholesterol transport by regulating ABCA1 expression^68,69^ and by regulating cell proliferation. Overexpression of miR-28-5p can inhibit the proliferation and migration of vascular smooth muscle cells in asymptomatic carotid artery stenosis patients.^70^ Our results complement these findings, as we observed that higher levels of miR-28-5p in EVs were correlated with decreased migration potential in endothelial monolayers following injury. Despite the evidence of miR-28-5p’s importance in cardiovascular pathologies, relatively little is known about its mechanism of action, especially in ECs, warranting further research. Innovative studies should investigate EV miR-28-5p expression and endothelial barrier function (e.g., flow-mediated dilation) in patients experiencing adverse SDoH in a PA intervention study to reduce CVD risk.

We acknowledge that our study is small and cross-sectional in nature, not allowing for causal inferences. Additionally, our small sample size, which comprised AA women study participants, does not allow for generalization to all AA individuals or individuals of other ethnicities and races, highlighting the need for larger, diverse studies incorporating a translational research component. Still, it highlights the importance of considering multi-level PA interventions that target both neighborhood-level deprivation and provide resources for increasing PA in individuals at the highest risk for CVD due to their lifelong exposure to adverse SDoH. Secondly, we acknowledge that utilizing ExoQuick to isolate EV from plasma samples may be seen as a limitation to this study, as it has been reported to include potential co-isolation of non-EV particles (e.g., lipoproteins). In this study, we cannot exclude the possibility that our study participants may have undergone rigorous PA right before they visited the NIH Clinical Center; however, this is unlikely given that fasting blood samples were done in the early morning for each participant.

Our study shows potential relationships between neighborhood deprivation as an adverse SDoH with EV size and cargo; our findings also demonstrate the potential mitigating associations between PA with EV size and cargo in a cohort of AA women at risk for CVD. While larger studies further evaluating these observations in diverse racial and ethnic groups are needed, our data suggest the importance of multi-level interventions to promote PA and improve neighborhood deprivation for patients residing in under-resourced communities. Future PA interventions in at-risk patients may reduce existing obesity-, diabetes- and CVD-related health disparities. EV content, concentration, and size may emerge as a mechanistic link or even as a therapeutic target. Future work should explore the associations discovered within this sample in a larger and more diverse cohort. Furthermore, we aim to expand this study to Step It Up, a place-tailored, community-engaged, digital health intervention^71^ clinical trial designed to increase PA, allowing for a significantly increased sample size and longitudinal data. Additionally, this interventional trial will allow for further analysis of additional measures of SDoH, immune cell distribution and function, and biomarkers of stress as well as the impact of place-tailored messaging on PA levels and EV characteristics.

## Methods

### Study design and participants

This study is focused on AA women living in lower-resourced neighborhoods within the Washington, DC metropolitan area (including the contiguous Prince George’s County, MD). These neighborhoods have a population with lower PA resources, lower median household income, are mainly AA, and contain the highest CVD burden compared to other neighborhoods in Washington, DC.^72^ A cohort of 24 AA women (Table 1) was recruited in collaboration with the DC Cardiovascular Health and Obesity Collaborative (DC CHOC) community advisory board to participate in pilot testing of Step It Up, a place-tailored digital app to increase PA in a community-engaged intervention.^71^ Eligibility included AA women between 21 and 75 years old who are overweight or obese, live in certain areas near Washington, DC and have a smartphone that can use the study app. Study approval was obtained from the Institutional Review Board (IRB) at the National Heart, Lung, and Blood Institute (NHLBI) at the National Institutes of Health (NIH) in accordance with the principles of the Declaration of Helsinki. The guidelines for good clinical practice and the Belmont Report (National Commission for the Protection of Human Subjects of Biomedical and Behavioral Research) were followed. Participants were enrolled under the IRB-approved clinical trial NCT03288207 (first registration date: 09/20/2017; https://clinicaltrials.gov/study/NCT03288207).^73^ All study participants provided written informed consent.

At baseline enrollment into the pilot intervention at the NIH Clinical Center (CC), participants underwent anthropometric and clinical laboratory measurements, cardiometabolic phenotyping, and physical examinations. Participant blood samples were collected in Heparin green-top tubes and processed for storage at -80 °C within 2 h after the fasting blood draw. Blood was drawn after an overnight fast between 8 and 9 am for all study participants recruited between October 2019 and March 2020. All participant samples were de-identified before distribution, and samples intended for storage were de-identified, labeled, and recorded in the Biospecimens Inventory System as recommended by NIH guidelines for human specimens.

### Exposures and covariates

NSD was calculated for each participant’s address at the 2010 census tract level and included the following variables: median household income; median home value; percent receiving welfare; percent below the poverty level; percent single mothers with children; percent households without a telephone; percent non-owner occupied units; percent households not receiving dividends, interest, or rental income; percent adults greater or equal to 25 years old without a high school diploma; percent adults greater or equal to 25 years old without a bachelor’s degree; and percent working adults not in an executive, managerial, or professional occupation.^74,75^ NSD was calculated as the sum of these variables, with a higher value representing a more deprived neighborhood.

Objective PA was determined in daily step counts. Participants received a wearable PA monitor (Fitbit Charge 2, Fitbit, San Francisco, CA) and were encouraged to wear it daily to track their steps. The Fitbit was chosen for its comfort, features, and ease of use, and it has been validated to reliably measure the outcome variables (steps per day) of this study.^72^.

Body mass index (BMI) was calculated from height and weight measurements. Atherosclerotic Cardiovascular Disease (ASCVD) 10-year risk score was calculated based on age, race, sex, total cholesterol, HDL cholesterol, blood pressure, and medical history, including diabetes and smoking status.^76,77^ The ASCVD risk score estimates a 10-year risk of experiencing an adverse CVD event.^77^ When adjusting for ASCVD, our cohort decreased to 22.

### Methods of measurements of study outcomes

#### Extracellular vesicle isolation and characterization

EVs were isolated from the fasting Heparin plasma samples (500 µl) using a precipitation method with ExoQuick^®^ ULTRA EV Isolation Kit for Serum and Plasma, according to the manufacturer’s instructions (System Biosciences, Palo Alto, CA). Heparin plasma samples were stored for up to two years at -80 °C before EV isolation. To determine their concentration and size distribution, EVs were diluted 1:100 in PBS, and nanoparticle tracking analysis (NTA) was performed using NanoSight 300 using the NTA software version 3.3 (https://www.malvernpanalytical.com/en/support/product-support/software/nanosight-nta-software-update-v3-4-4). The samples were loaded via automatic syringe at a standard flow rate consistent for all of the samples (Camera Level: 13–15, Screen Gain: 1, Capture Number: 3, Capture Time Length: 10 s, Temperature: 25 °C), as previously described.^78,79^ Dilution factor (1:100) was taken into account through the input settings of the NanoSight instrument. For both the EV size and concentration, we reported the average values that were generated by the NanoSight software. EV morphology and size were confirmed using negative staining electron microscopy. EVs were resuspended in sterile water in order to prepare samples for electron microscopy (EM). A drop of EV suspension was mixed with an equal volume of 1% phosphotungstic acid solution on the surface of a formvar-coated carbonated copper grid. The excess fluid was removed from the grid and imaging was performed using a Jeol J100 electron microscope. The EV morphology was consistent with a lipid bound, donut-shape structure with average diameters consistent in size with ones obtained using the nanotracking analyzer. Unfortunately, the limited amount of sample available prevented us from performing western blots of the EV markers, as specified by the MISEV guidelines [15]. However, we previously reported EV protein markers confirmed via Western blotting, LC/MS/MS^79^ or ExoView chips^78^. The MIBlood-EV document is attached as a supplementary file.

For a set of in vitro experiments utilizing isolated EVs from study participants, 12 of 24 samples were chosen based on EV size and concentration differences. For this, the data of all study participants were sorted by EV size after EV size was determined to be the main outcome and finding of Fig. 1. The upper and lower quantiles (*n* = 6) based on size rankings were selected for subsequent analysis of miRNA content.

#### In vitro endothelial cell culture and barrier function measurement

Human Coronary Artery Endothelial Cells (HCAECs) were purchased from Cell Applications Inc. (San Diego, CA), cultured and expanded in MesoEndo Cell Growth media, according to manufacturer’s instructions, and used in experiments between passages 4–8. Approximately 50,000 to 75,000 cells were seeded in each well and let grow overnight. The experiment was conducted when a stable resistance was achieved above 800Ω, indicative of a confluent monolayer.^78^ At that point, HCAECs were treated with 50,000 EV/cell or left untreated as a control and incubated for 1 h before starting the recording. This EV concentration was previously used in our lab and was shown to affect HCAEC permeability and migration significantly.^78,79^ For the wound healing experiments, cells were wounded through pulses of 3000 µAmps, and resistance was measured for the following 48 h to generate data representing HCAEC migration. Experiments were performed in triplicates and repeated three times. Resistance values were collected and normalized to each well’s value at t = 0. The area under the curve (AUC) was calculated from the graphs generated using this normalized data and used to determine statistical associations. HCAECs were subjected to the Electric Cell-Substrate Impedance Sensing (ECIS) technique using an ECIS model 1600R ζθ instrument (Applied Biophysics) on the 96-well gold electrode arrays (96W10idf) to measure endothelial barrier integrity.

#### miRNA analysis of extracellular vesicles

EV total RNA was extracted as previously described using Norgen Single Cell RNA Purification Kit (Biotek Corporation, Calabasas, CA, catalog no. 51800).^78^ The quality and concentration of the isolation were determined using a NanoDrop spectrophotometer. EV RNA was analyzed for the expression of 828 miRNAs using a Human v3 miRNA panel (Nanostring Technologies, Seattle, WA, United States). The panel includes five non-mammalian spike-in miRNA probes as negative controls and five internal reference genes used to normalize data. Raw data were quality control assessed and normalized by the NanoString nSolver™ analysis and Rosalind Bio software. Rosalind quality control of NanoString miRNA data was performed by assessing the total number of fields of view captured and the binding density of barcode capture by spots per square micron. The noise threshold was set by calculating the number of standard deviations between the average negative control value and the positive control. Ligation control threshold was calculated by determining the number of standard deviations of the positive controls that are above the negative controls. Normalization of miRNA data was performed by Rosalind software (version 3.16, https://www.rosalind.bio/en/knowledge/nanostring-mirna-rcc-methods). Normalization consisted of a two-step data transformation involving a positive control r and a codeset normalization factor. Both normalization factors were generated by calculating the geometric mean of the selected probes, then the arithmetic means of those geometric means for all samples. The normalization factor, the ratio of the arithmetic mean vs. the geometric mean, was then multiplied by the counts for every probe by its lane-specific normalization factor. Following normalization, any miRNA that was below a detection threshold of 20 counts was considered undetectable and excluded from any downstream analysis. The detection threshold of 20 counts was used, as recommended by the manufacturer.

The miRNAs that significantly differed by size, concentration, and step count were further examined for their influence on biological pathways. The miRNA gene targets were obtained from the miRPathDBv2.0 database.^80^ The individual as well as the miRNA-shared gene targets were used for overrepresentation analysis against the Reactome pathway database with Webgestalt (https://www.webgestalt.org/) to identify pathways significant at FDR ≤ 0.05.

### Statistical analysis

Since this cohort was at risk for CVD, it was important to determine associations beyond CVD risk factors. Associations between NSD, PA, and EV were analyzed using multivariable regression analysis adjusted for body mass index (BMI) and atherosclerotic cardiovascular disease (ASCVD) 10-year risk score. STATA (StataCorp. Version 17. College Station, TX, USA: StataCorp LLC.; www.stata.com) was used for all regression analyses. P-values were adjusted for multiple comparisons utilizing R software (version 4.4.0; https://www.r-project.org/). False discovery rate (FDR) was performed for multiple comparisons of p-values^81^(raw p-values are indicated by p, while FDR adjusted p-values are indicated with FDR p). Using GraphPad PRISM 10 software (version 10.4.2, https://www.graphpad.com/features), a bivariate comparison was made for miRNA content differences by concentration and size. The dataset’s normality was tested first to determine subsequent analysis (unpaired t-test vs. Mann-Whitney test). Additionally, depending on the dataset’s distribution, endothelial barrier function and miRNA content were subjected to correlation analysis (Spearman correlation). Individual graphs were visualized using GraphPad PRISM 10 software, while the layout of all Figures was done in Adobe Photoshop 2024 (version 25; www.adobe.com).

## Supplementary Information

Below is the link to the electronic supplementary material.

Supplementary Material 1

Supplementary Material 2

## Data Availability

The datasets generated and analyzed during the current study are available from the corresponding author upon reasonable request.
