# Supplementary material for: Neighborhood socioeconomic deprivation and physical activity associate with extracellular vesicle size and cargo in a community-based cohort of women
Source: Sci Rep. 2026 Apr 28;16:19804. doi: 10.1038/s41598-026-49679-6 (PMC13315576; doi:10.1038/s41598-026-49679-6)
Supplement: Supplementary file 1 — Supplementary Material 1 [file 41598_2026_49679_MOESM1_ESM.pdf]

INTERNATIONAL SOCIETY for EXTRACELLULAR VESICLES

MIBlood-EV

Standardized Reporting Tool for Blood EV Research (Human)

STUDY INFORMATION

1.0 **Manuscript title**	Physical Activity, Socioeconomic Deprivation and Extracellular Vesicles in At-Risk African American Women: Insights from the Step It Up Pilot Study		
1.1 **Corresponding author (Name and Email)**	Dr. Tiffany Powell-Wiley tiffany.powell-wiley@nih.gov		
1.2 **Institution name**	National Heart, Lung and Blood Institute		
1.3 **Time period of experiment (e.g. 2022-2024)**	2021-2023	1.4 **Number of samples**	24
1.5 **Cargo of interest**	☑ Vesicles ☐ Protein ☐ RNA ☐ DNA ☑ Other: miRNA		
1.6 **Biospecimen type**	☑ Plasma ☐ Serum	1.7 **Biospecimen state**	Frozen
1.8 **Source of frozen specimens**	Prepared by the lab	1.9 **Years of collection (range)**	2

BLOOD COLLECTION AND PROCESSING

2.0 **Patient fasting status**	Fasted	2.1 **Fasting length (e.g. hours/days)**	8-10 hours
2.2 **Anatomical access site**	Anterior cubital vein	2.3 **Needle diameter (e.g. gauge)**	21-22 gauge
2.4 **Blood volume collected (mL)**	8 mL		
2.5 **Plasma anticoagulant**	☐ EDTA ☐ Citrate ☑ Heparin ☐ Other:		
2.6 **Serum tube type**		2.7 **Serum clotting time (minutes)**	
2.8 **Time between collection and first centrifugation (range in hours)**	1-2 hours		
2.9 **Transport temperature**	Room temp (~22°C)	2.10 **Transport condition of tubes**	Vertical
2.11 **Centrifuge brand and model**	HErmle Benchmark Z326K		
2.12 **Bucket rotor type**	Swing	2.13 **Number of centrifugation cycles**	One
2.14a **1st Centrifugation speed (RCF in x g)**	1,200	2.14b **1st Centrifugation time (minutes)**	20
2.15 **1st Rotor brake**	On	2.16 **1st Centrifugation temperature**	4°C
2.17a **2nd Centrifugation speed (RCF in x g)**		2.17b **2nd Centrifugation time (minutes)**	
2.18 **2nd Rotor brake**		2.19 **2nd Centrifugation temperature**	
2.20 **Additional processing steps (e.g. filtration)**	None		
2.21 **Storage tubes (brand, type, source, catalog number)**	Freestanding microcentrifuge Tubes with flat screw caps 0.5ml; VWR; 16466-052		
2.22 **Storage temperature**	-80°C	2.23 **Length of storage (range in years)**	1-2 years

PLASMA/SERUM QUALITY CONTROL

3.0 **Number of freeze-thaw cycles (range)**	1		
3.1 **Thawing temperature**	on ice	3.2 **Thawing duration (minutes)**	~60min

Hemolysis

3.3 **Presence of hemolysis**	Yes	3.4 **Frequency of hemolyzed samples (e.g. <25%, 25-50%)**	0%
3.5 **Method used**	Visual inspection	3.6 **RBC count (Median, 95% CI, N)**	
3.7 **RBC counter brand and type**			
3.8 **Spectrophotometry hemoglobin concentration (mean g/L)**			
3.9 **Spectrophotometer brand, model and wavelength measured (e.g. 414 nm)**			
3.10 **Hemolized samples were discarded**	Yes		

Platelets

3.11 Presence of platelets	Not Tested	3.12 Method used (e.g. Flow Cytometry)	
3.13 Marker(s) used (e.g. CD61, CD41)			
3.14 Concentration (median, 95% CI, N)			
3.15 Platelet counter instrument brand, type and limit of detection (cells/L)			
3.16 Flow cytometer brand and type			
3.17 Flow cytometry size and fluorescence ranges of detection in nanometers and MESF, respectively			

Lipoproteins

3.18 Presence of lipoproteins	Not tested	3.19 Method used (WB, ELISA, FC)	
3.20 Spectrophotometry L-index			
3.21 Spectrophotometer brand, model and wavelength measured (e.g. 700 nm)			
3.22 WB Marker(s) used (e.g. Apo B)			
3.23 Western blot images provided in manuscript?			
3.24 Flow cytometry marker(s) used (e.g. ApoB)			
3.25 Flow cytometry concentration (median, 95% CI, N)			
3.26 Flow cytometer brand and type			
3.27 Flow cytometry size and fluorescence ranges of detection in nanometers and MESF, respectively			
3.28 Brand and catalog number for ELISA kit used for each Apolipoprotein test (e.g. ApoB)			
3.29 Concentration measured for each Apolipoprotein tested (mean µg/mL)			
