# Supplementary material for: Neighborhood socioeconomic deprivation and physical activity associate with extracellular vesicle size and cargo in a community-based cohort of women
Source: Sci Rep. 2026 Apr 28;16:19804. doi: 10.1038/s41598-026-49679-6 (PMC13315576; doi:10.1038/s41598-026-49679-6)
Supplement: Supplementary file 2 — Supplementary Material 2 [file 41598_2026_49679_MOESM2_ESM.docx]

**Title:**

Neighborhood Socioeconomic Deprivation, and Physical Activity Associate with Extracellular Vesicle Size and Cargo in a Community-Based Cohort of Women

**Authors**:

Yvonne Baumer^1#^, Elizabeth M. Aquino Peterson^1#^, Matthew R. Bavuso^2^, Noel Miller^2^, Cristhian A. Gutierrez-Huerta^1^, Nithya P. Vijayakumar^1^, Sam J. Neally^1^, Kaveri Curlin^1^, Valerie M. Mitchell^1^, Billy S. Collins^1^, Neelam R. Redekar^3^, Subrata Paul^3^, Anca Dobrian^2†^, Tiffany M. Powell-Wiley^1,4†*^

^#^ These two authors contributed equally to this manuscript.

^†^Shared senior authors

^*^ Corresponding author

^1^ Social Determinants of Obesity and Cardiovascular Risk Laboratory, National Heart Lung and Blood Institute, National Institutes of Health, Bethesda, MD, USA.

^2^ Department of Biomedical and Translational Sciences, Eastern Virginia Medical School at Old Dominion University, Norfolk, VA, USA

^3^ Integrative Data Sciences Section, National Institute of Allergy and Infectious Diseases, National Institutes of Health, Bethesda, MD, USA

^4^ Intramural Research Program, National Institute on Minority Health and Health Disparities, National Institutes of Health, Bethesda, MD, USA.

**Corresponding author:** Chief, Social Determinants of Obesity and Cardiovascular Risk Laboratory, National Heart, Lung, and Blood Institute, Adjunct Investigator, Intramural Research Program, National Institute on Minority Health and Health Disparities, Building 10-CRC, Room 5-5332, Bethesda, MD 20892, USA. E-mail address: tiffany.powell-wiley@nih.gov (T.M. Powell-Wiley).

**Supplement**

**Supplementary Figures**


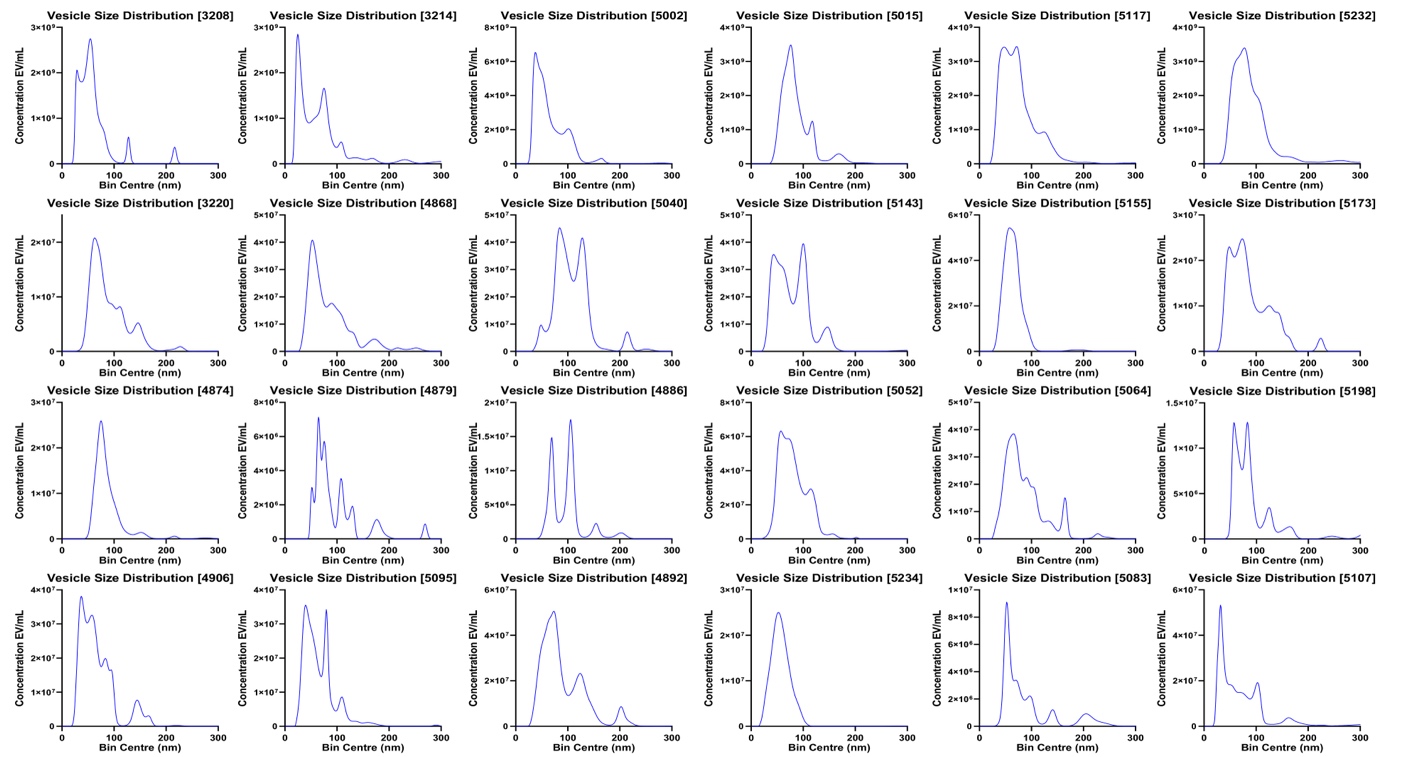


**Supplementary Figure 1:** Display of the size distribution profile of each donors’ EVs.


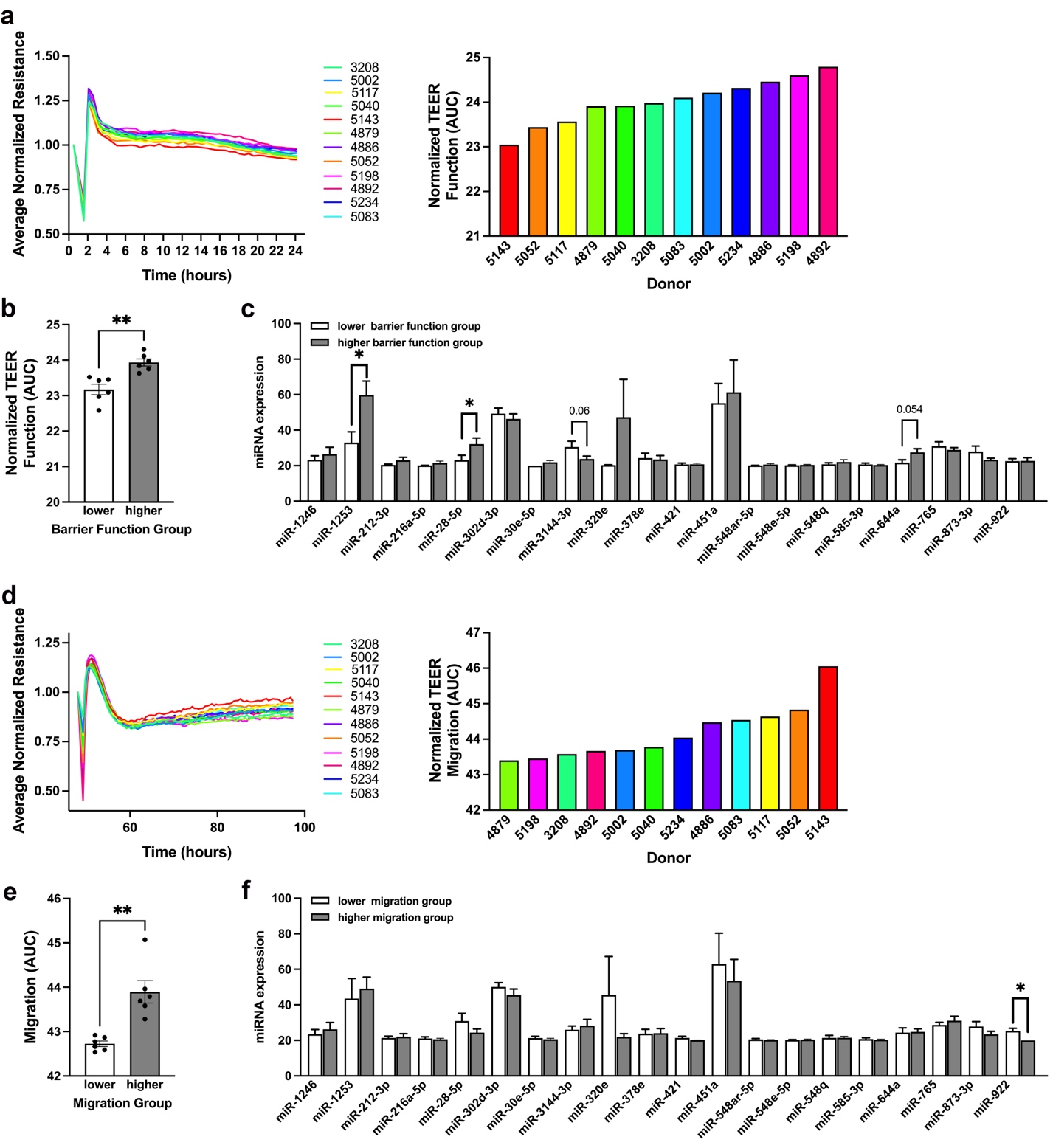


**Supplementary Figure 2: MiR differences in EVs as bivariate comparison by endothelial barrier integrity or EC migration.** (**a**) Display of the resistance curves for HCAECs treated with EVs of one of the study participants (de-identified 4-digit numbers). Area under the curve (AUC) was determined for each donor and is displayed as a bar graph. (**b**) Separating the observed AUCs by their extremes is displayed by a bar graph. (n=6 each group, significance is indicated by the asterix * after the p-value is <0.05). (**c**) Bivariate comparison of EV miR content by barrier function AUC. (**d**) Display of the resistance curves for HCAECs treated with EVs of one of the study participants (de-identified 4-digit numbers) after wounding. Area under the curve (AUC) after wounding was determined for each donor and is displayed as a bar graph. (**e**) Separating the observed AUCs by their extremes of migration AUC is displayed by a bar graph. (n=6 each group, significance is indicated by the asterix * after the p-value is <0.05). (**f**) Bivariate comparison of EV miR content by EC migration AUC. [Significance is indicated by the asterix * when the p<0.05 was calculated; ** indicates significance with a p-value < 0.001; p-values indicate raw unadjusted p-values; AUC-area under the curve]


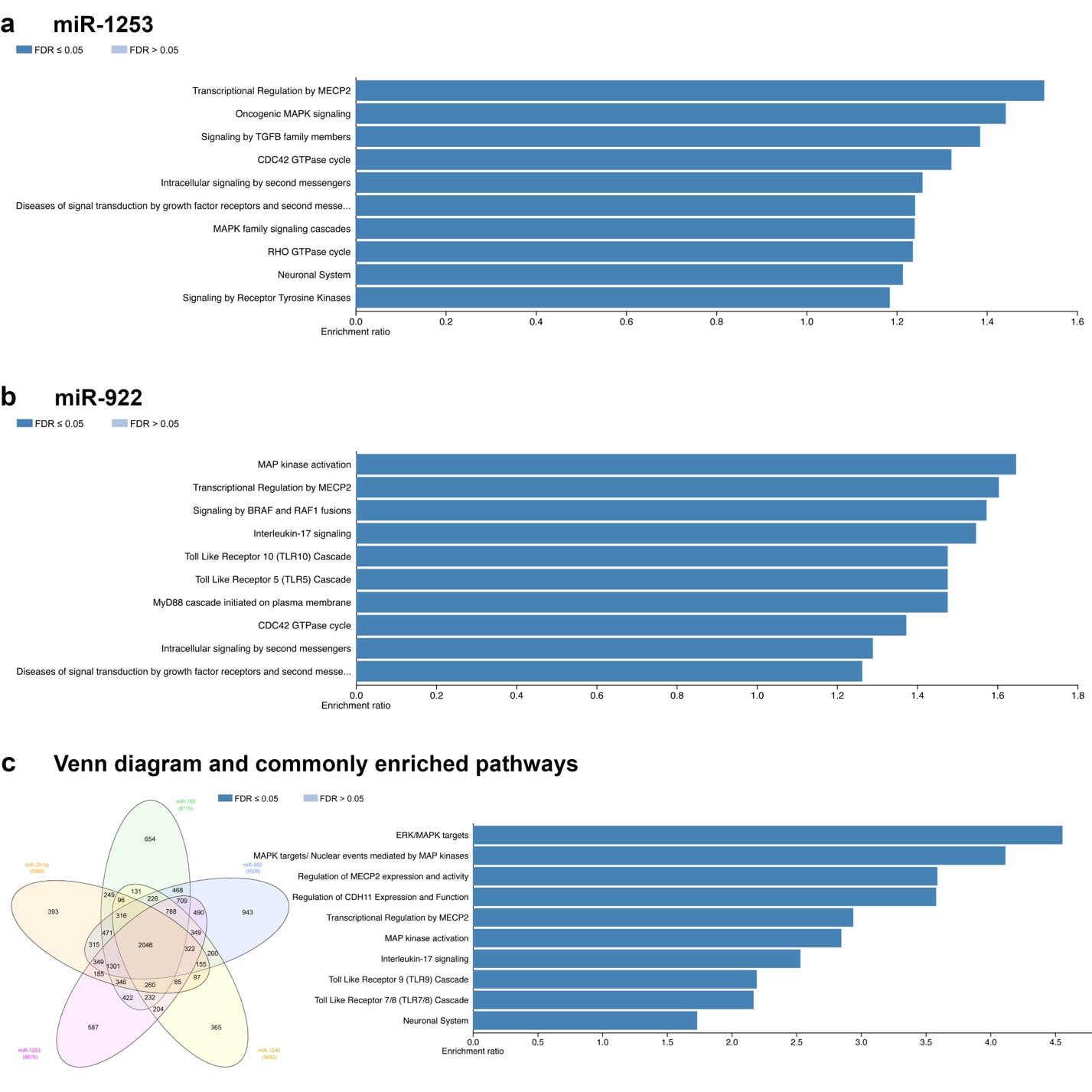


**Supplementary Figure 3:** **Bar graph representation of signaling pathways shown to be significantly impacted by the indicated miRNAs in an unbiased biostatistical analysis approach.** The two miRNAs shown to be significantly different in EVs by either endothelial barrier function or endothelial migration potential are miR-1253 (**a**) and miR-922 (**b**). The x-axis indicates the enrichment ratio, while the y-axis indicates the signaling pathway identified and sorted by the database used. (**c**) Pathway enrichment of shared gene targets of miR-1246, miR-1253, miR-28-5p, miR-765, miR-922 microRNAs. The left panel displays the Venn diagram showing the intersection of the miRNA gene targets. The right panel shows the Reactome pathway enrichments using common gene targets of three miRNAs. The x-axis indicates the enrichment ratio, while the y-axis indicates the significantly enriched Reactome pathways.

**Supplementary Tables:**

**Supplementary Table 1:** Enrichment analysis of miRNAs found to be significantly differently expressed in EVs isolated from patients. Listed are significant Reactome pathways with an FDR ≤ 0.05.

| **miRNA** | **Pathway** | **Size** | **Overlap** | **Expect** | **Ratio** | **p-value** | **FDR p-value** |
| --- | --- | --- | --- | --- | --- | --- | --- |
| miR-1246 | Gene expression (Transcription) | 1543 | 587 | 483.965755 | 1.21289573 | 1.0833E-09 | 2.1525E-06 |
|  | Generic Transcription Pathway | 1251 | 482 | 392.379235 | 1.22840343 | 6.6623E-09 | 6.619E-06 |
|  | RNA Polymerase II Transcription | 1375 | 523 | 431.272141 | 1.21269136 | 1.1363E-08 | 7.5264E-06 |
|  | Neuronal System | 410 | 174 | 128.597511 | 1.35305885 | 9.8038E-07 | 0.00048701 |
|  | MAPK targets/ Nuclear events mediated by MAP kinases | 31 | 22 | 9.72322645 | 2.26262343 | 6.6793E-06 | 0.00265434 |
|  | Signaling by Receptor Tyrosine Kinases | 526 | 208 | 164.981197 | 1.26074973 | 2.8807E-05 | 0.00953976 |
|  | Activation of NMDA receptors and postsynaptic events | 93 | 47 | 29.1696794 | 1.61126214 | 8.6168E-05 | 0.02445928 |
|  | Post NMDA receptor activation events | 80 | 41 | 25.0921973 | 1.63397408 | 0.00016106 | 0.04000223 |
|  | Transmission across Chemical Synapses | 270 | 112 | 84.6861659 | 1.32253006 | 0.00024503 | 0.04397748 |
|  | Signaling by EGFR | 50 | 28 | 15.6826233 | 1.78541558 | 0.00026041 | 0.04397748 |
|  |  |  |  |  |  |  |  |
| miR-28-5p | Neuronal System | 410 | 204 | 150.831138 | 1.35250587 | 3.2048E-08 | 6.368E-05 |
|  | Transmission across Chemical Synapses | 270 | 140 | 99.3278227 | 1.40947417 | 2.33E-07 | 0.00023148 |
|  | CREB1 phosphorylation through NMDA receptor-mediated activation of RAS signaling | 29 | 24 | 10.6685439 | 2.24960409 | 4.9341E-07 | 0.0003268 |
|  | Membrane Trafficking | 634 | 286 | 233.236443 | 1.22622347 | 5.8414E-06 | 0.00290171 |
|  | CDC42 GTPase cycle | 155 | 83 | 57.0215278 | 1.4555906 | 1.3803E-05 | 0.00535567 |
|  | Vesicle-mediated transport | 673 | 299 | 247.583795 | 1.20767193 | 1.6172E-05 | 0.00535567 |
|  | Activation of NMDA receptors and postsynaptic events | 93 | 54 | 34.2129167 | 1.57835125 | 2.2862E-05 | 0.00648968 |
|  | Neurotransmitter receptors and postsynaptic signal transmission | 205 | 104 | 75.4155691 | 1.37902559 | 2.7165E-05 | 0.00674704 |
|  | Ras activation upon Ca2+ influx through NMDA receptor | 21 | 17 | 7.72549732 | 2.20050558 | 4.4464E-05 | 0.0098166 |
|  | Transcriptional Regulation by MECP2 | 62 | 38 | 22.8086111 | 1.66603744 | 7.3785E-05 | 0.01466107 |
|  |  |  |  |  |  |  |  |
| miR-765 | Neuronal System | 410 | 240 | 187.552003 | 1.27964509 | 8.0503E-08 | 0.00015996 |
|  | MAPK family signaling cascades | 325 | 191 | 148.669271 | 1.28473086 | 1.1784E-06 | 0.00087777 |
|  | Transcriptional Regulation by MECP2 | 62 | 47 | 28.3615224 | 1.65717479 | 1.3253E-06 | 0.00087777 |
|  | Regulation of MECP2 expression and activity | 33 | 28 | 15.095649 | 1.8548391 | 3.8886E-06 | 0.00174861 |
|  | Transmission across Chemical Synapses | 270 | 160 | 123.509856 | 1.29544318 | 4.4001E-06 | 0.00174861 |
|  | Transport of small molecules | 727 | 388 | 332.561722 | 1.16670072 | 1.2042E-05 | 0.00322104 |
|  | Oncogenic MAPK signaling | 84 | 58 | 38.4252884 | 1.50942263 | 1.266E-05 | 0.00322104 |
|  | Neurexins and neuroligins | 55 | 41 | 25.159415 | 1.62960864 | 1.2968E-05 | 0.00322104 |
|  | CREB1 phosphorylation through NMDA receptor-mediated activation of RAS signaling | 29 | 24 | 13.2658734 | 1.80915341 | 4.5987E-05 | 0.01015299 |
|  | Glycosaminoglycan metabolism | 126 | 79 | 57.6379326 | 1.37062515 | 8.792E-05 | 0.01626341 |

**Supplementary Table 2:** Enrichment analysis of miRNAs found to be significantly differently expressed in EVs isolated from patients. Listed are significant Reactome pathways with an FDR ≤ 0.05.

| **miRNA** | **Pathway** | **Size** | **Overlap** | **Expect** | **Ratio** | **p-value** | **FDR p-value** |
| --- | --- | --- | --- | --- | --- | --- | --- |
| miR-1253 | Diseases of signal transduction by growth factor receptors and second messengers | 433 | 244 | 196.617949 | 1.24098538 | 2.0588E-06 | 0.00213089 |
|  | RHO GTPase cycle | 449 | 252 | 203.883277 | 1.23600132 | 2.1448E-06 | 0.00213089 |
|  | Intracellular signaling by second messengers | 310 | 177 | 140.765737 | 1.25740826 | 1.8533E-05 | 0.01227473 |
|  | Neuronal System | 410 | 226 | 186.174039 | 1.21391791 | 3.6621E-05 | 0.01541877 |
|  | MAPK family signaling cascades | 325 | 183 | 147.576982 | 1.24003078 | 4.0785E-05 | 0.01541877 |
|  | Signaling by Receptor Tyrosine Kinases | 526 | 283 | 238.84767 | 1.1848556 | 4.6559E-05 | 0.01541877 |
|  | Signaling by TGFB family members | 124 | 78 | 56.3062948 | 1.38528028 | 6.1033E-05 | 0.01732476 |
|  | Transcriptional Regulation by MECP2 | 62 | 43 | 28.1531474 | 1.52736031 | 0.00011503 | 0.0285708 |
|  | Oncogenic MAPK signaling | 84 | 55 | 38.1429739 | 1.44194315 | 0.00015849 | 0.03322175 |
|  | CDC42 GTPase cycle | 155 | 93 | 70.3828686 | 1.32134427 | 0.0001672 | 0.03322175 |
|  |  |  |  |  |  |  |  |
| miR-922 | Diseases of signal transduction by growth factor receptors and second messengers | 433 | 275 | 217.738941 | 1.26298033 | 1.0763E-08 | 2.1386E-05 |
|  | MAP kinase activation | 64 | 53 | 32.1831229 | 1.64682589 | 5.9249E-08 | 5.8863E-05 |
|  | Intracellular signaling by second messengers | 310 | 201 | 155.887002 | 1.28939551 | 1.1234E-07 | 7.4407E-05 |
|  | Transcriptional Regulation by MECP2 | 62 | 50 | 31.1774003 | 1.60372576 | 7.0953E-07 | 0.00028363 |
|  | Signaling by BRAF and RAF1 fusions | 67 | 53 | 33.6917068 | 1.57308742 | 1.0385E-06 | 0.00028363 |
|  | Toll Like Receptor 10 (TLR10) Cascade | 97 | 72 | 48.7775456 | 1.47608903 | 1.1419E-06 | 0.00028363 |
|  | Toll Like Receptor 5 (TLR5) Cascade | 97 | 72 | 48.7775456 | 1.47608903 | 1.1419E-06 | 0.00028363 |
|  | MyD88 cascade initiated on plasma membrane | 97 | 72 | 48.7775456 | 1.47608903 | 1.1419E-06 | 0.00028363 |
|  | Interleukin-17 signaling | 72 | 56 | 36.2060133 | 1.5467044 | 1.4077E-06 | 0.00029182 |
|  | CDC42 GTPase cycle | 155 | 107 | 77.9435008 | 1.37278925 | 1.4686E-06 | 0.00029182 |

**Supplementary Table 3:** Enrichment analysis of shared gene targets of miRNAs found to be significantly differently expressed in EVs isolated from patients. Listed are pathways are significant at FDR <= 0.05.

| **Pathway** | **Size** | **Overlap** | **Expect** | **Ratio** | **p-value** | **FDR p-value** |
| --- | --- | --- | --- | --- | --- | --- |
| Neuronal System | 410 | 78 | 44.98865 | 1.733771 | 6.39E-07 | 0.00127 |
| MAPK targets/ Nuclear events mediated by MAP kinases | 31 | 14 | 3.401581 | 4.115734 | 1.49E-06 | 0.001479 |
| Transcriptional Regulation by MECP2 | 62 | 20 | 6.803161 | 2.93981 | 5.45E-06 | 0.002953 |
| ERK/MAPK targets | 22 | 11 | 2.414025 | 4.556705 | 5.94E-06 | 0.002953 |
| MAP kinase activation | 64 | 20 | 7.022618 | 2.847941 | 9.37E-06 | 0.003724 |
| Regulation of MECP2 expression and activity | 33 | 13 | 3.621037 | 3.590131 | 2.18E-05 | 0.007219 |
| Interleukin-17 signaling | 72 | 20 | 7.900445 | 2.531503 | 6.32E-05 | 0.017932 |
| Toll Like Receptor 9 (TLR9) Cascade | 108 | 26 | 11.85067 | 2.193969 | 8.05E-05 | 0.019995 |
| Regulation of CDH11 Expression and Function | 28 | 11 | 3.072395 | 3.580268 | 9.7E-05 | 0.021413 |
| Toll Like Receptor 7/8 (TLR7/8) Cascade | 105 | 25 | 11.52148 | 2.16986 | 0.000133 | 0.024036 |
